# Feature and Contrast Enhancement of Mammographic Image Based on Multiscale Analysis and Morphology

**DOI:** 10.1155/2013/716948

**Published:** 2013-12-12

**Authors:** Shibin Wu, Shaode Yu, Yuhan Yang, Yaoqin Xie

**Affiliations:** ^1^Shenzhen Institute of Advanced Technology, Chinese Academy of Science, China; ^2^Shenzhen Key Laboratory for Low-Cost Healthcare, China

## Abstract

A new algorithm for feature and contrast enhancement of mammographic images is proposed in this paper. The approach bases on multiscale transform and mathematical morphology. First of all, the Laplacian Gaussian pyramid operator is applied to transform the mammography into different scale subband images. In addition, the detail or high frequency subimages are equalized by contrast limited adaptive histogram equalization (CLAHE) and low-pass subimages are processed by mathematical morphology. Finally, the enhanced image of feature and contrast is reconstructed from the Laplacian Gaussian pyramid coefficients modified at one or more levels by contrast limited adaptive histogram equalization and mathematical morphology, respectively. The enhanced image is processed by global nonlinear operator. The experimental results show that the presented algorithm is effective for feature and contrast enhancement of mammogram. The performance evaluation of the proposed algorithm is measured by contrast evaluation criterion for image, signal-noise-ratio (SNR), and contrast improvement index (CII).

## 1. Introduction

Breast cancer has been a significant public health problem for women in the world and early detection of breast cancer is very essential in the field of medicine before the means to prevent breast cancer have not yet been found. However, there are new cases 234580 and death rate 17.1% from the National Cancer Institute in the United States in 2013 [1]. Breast cancer accounted for about more than 38% of cancer incidence and a significant percentage of cancer mortality in the developing and developed countries in 2009 [2]. Thus, it is well known that the early detection and treatment of breast cancer are the most effective key means of reducing mortality. Furthermore, mammography is widely recognized as being the only effective and primary imaging modality for the early detection and diagnosis of breast cancer [3–5]. In mammography, low dose X-ray is used for imaging. Hence, the mammographic images are poor in contrast and contaminated due to the low dose X-ray for imaging. In low contrast mammograms, it is difficult to interpret between the normal tissue and malignant tissue. In addition, [6] introduced that mammographers miss about 10% of all cancerous lesions when using the poor contrast mammograms.

In recent years, there are many researchers that proposed all kinds of contrast enhancement algorithms to solve these problems produced by poor contrast images. Sundaram et al. [6] proposed the contrast enhancement method based on histogram to improve the mammography quality, but this method has not suppressed the amplified noise in histogram equalization progress. Mohideen et al. [2] used multiwavelet with hard threshold to denoise and enhance mammographic image contrast. Kumar et al. [7] proposed the algorithm based on morphology and wavelet transform for enhancement of mammographic images. Morrow et al. [8] designed a region-based contrast enhancement algorithm for mammograms. This method uses each pixel in the image as a seed to grow a region. Contrast is then enhanced by applying an empirical transformation based on each region's seed pixel value, its contrast and background information. Stojić et al. [9] developed an algorithm using mathematical morphology to enhance local contrast of mammography. Stahl et al. [10] applied the method of nonlinear multiscale processing based on Laplace pyramid for digital radiography enhancement. However, the binomial filter was used at each pyramid level with a nonlinear factor for contrast enhancement, which was sensitive to noise. Comparing with Stahl's algorithm, Gaussian filter is used in the proposed method. As far as we know, most essential image processing algorithms applied the Gaussian filter, while the binomial filter applications are not popular in state-of-the-art image processing algorithms. Multiscale analysis technology based on the wavelet transform for mammogram contrast enhancement was applied in [2, 7, 11–15]. However, the wavelet transform leads to undesirable artifacts in the enhanced image [16]. To overcome this shortcoming, we propose an algorithm framework, in which the wavelet transform is replaced with the Laplacian Gaussian pyramid transform for multiscale analysis. Comparing with the wavelet technique, the Laplacian Gaussian pyramid technique seems to be a more suitable decomposition method for multiscale contrast enhancement of mammogram [16].

We compare the suitability of these methods for enhancement of mammographic images in general. We propose the approach which seems to be suitable for contrast enhancement of mammograms. Traditional multiscale analysis methods only enhance the high frequency components of mammogram. The proposed approach decomposes the image by the Laplacian Gaussian pyramid transform, firstly. Secondly, the CLAHE is adopted to enhance contrast and detail information of each level high frequency subimage decomposed by Laplacian pyramid transform and Gaussian filter is used to restrain the CLAHE to enlarge the noise of each subimage. Furthermore, mathematical morphology is applied to enhance local features and contrast at each pyramid level's subimage. Finally, the inverse Laplacian Gaussian pyramid transform is applied to reconstruct the decomposed subimages to obtain the features and contrast enhancement image. The enhanced image was adjusted the entire contrast by a global non-linear operator for nature visualization.

The rest of this paper is organized as follows. Theory of the Laplacian Gaussian pyramid transform and the key characteristics of the CLAHE and mathematical morphology are detailed in Section 2.  Section 3 presents the experimental results and discussion. The conclusion of this paper is stated in Section 4.

## 2. Materials and Method

### 2.1. The Proposed Method

Traditional image enhancement techniques cannot adapt to the varying characteristic of images. The application of a global transform or a fixed operator to an entire image often yields poor result at least in some parts of the given image [10]. In order to solve these problems, we propose a novel approach based on the multiscale analysis and mathematical morphology, which cannot only enhance the local detail information and edges, but also restrict the modified noise effectively.

To begin with, the original image is decomposed by the Laplacian Gaussian pyramid transform to obtain low frequency subband images and different scale coefficients of the high frequency components. At present, all kinds of filter techniques, including gray-scale operator, median filtering, edge enhancement operator, frequency enhancement operator, spatial-frequency filtering, and anisotropic adaptive filtering, are applied to reduce the noises of image for achieving image enhancement. However, these techniques are suitable for features enhancement of the whole image. In fact, we do need to enhance the local features and edges of medical image in some practical clinical application. Mathematical morphology operations fit these requirements. The morphology operators can enrich the low frequency information of the image and enhance this part contrast in terms of the principle of mathematical morphology. Therefore, noise in different multiscale subimages can be suppressed by morphology operations. Vessels, fibroglandular tissues, masses, and calcification points are reinforced at each pyramid level. Furthermore, morphology operators can make the feature details become smoother and the edges sharper. Most essential image processing algorithm can be represented in the form of morphological operations. In addition, the low frequency subbands contain basic information and parts contrast of image. Therefore, the processing of low frequency data is important and cannot be ignored. For the low-pass filtered subband images, we apply the mathematical morphological operations, combining opening operation with closing operation, to enrich the basic information and enhance image contrast.

Secondly, histogram equalization is used for enriching detail information and sharping edges of the whole image. The detail information and image edges belong to the high frequency components. Furthermore, the high frequency subbands contain noises. We adopt the CLAHE to enhance the high frequency subbands coefficients, which can not only enhance the features and image contrast and enrich the detail information and image edges but also effectively suppresses the enlarged noise. Comparing with the AHE, the CLAHE can reduce computational time and effectively suppress the enlarged noise. Comparing with the traditional histogram equalization and the modified histogram equalization, the CLAHE can effectively enhance the local features, edges, and image contrast.

**Figure 1:**
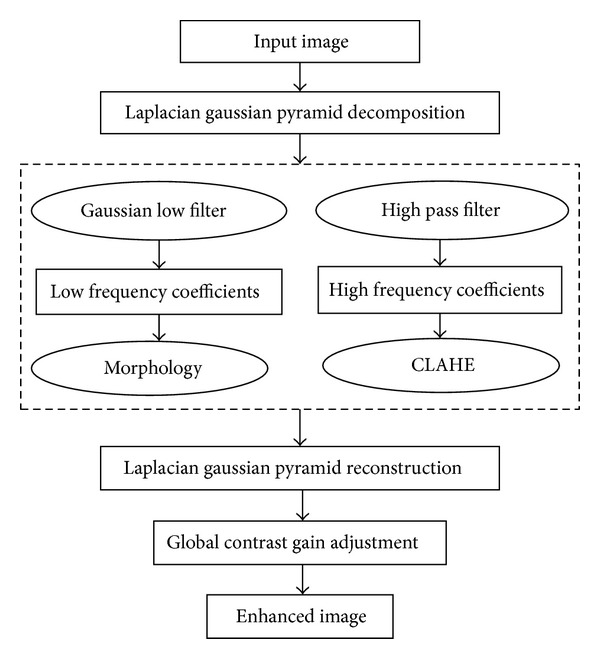
Flowchart of the proposed method.

Finally, we can reconstruct an enhanced image, and its size is the same as the original image. We apply the contrast low frequency coefficients adjusted through mathematical morphology and the high frequency subbands processed by the CLAHE to extract the enhanced feature and contrast image due to the Laplacian Gaussian pyramid transform is of the property of reversal. Besides, a global gain operation is used to adjust the contrast of reconstructed image in order to make the enhanced image more natural and smoother. The flowchart of the proposed algorithm is shown in Figure 1.

### 2.2. The Laplacian Gaussian Pyramid Transform

**Figure 2:**
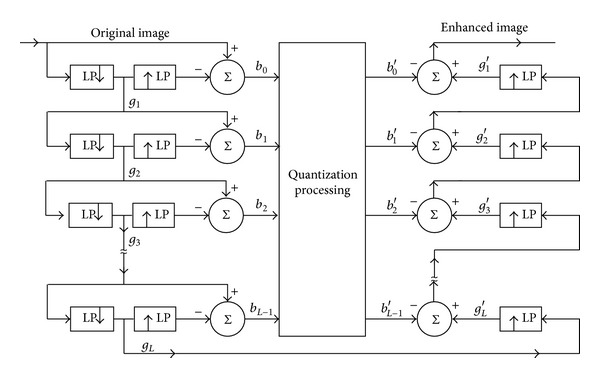
Flow diagram of the Laplacian Gaussian pyramid transform.

The Laplacian Gaussian pyramid technique was developed by Burt and Adelson for the context of compression of images [16, 17]. The Laplacian Gaussian pyramid transform has been used to analyze images at multiscale analysis for a broad range of application [17]. The purpose of multiscale image contrast enhancement is that the original image is divided into several different multiscale levels' subband images which are enhanced by the CLAHE and morphology operations, respectively. All levels' subband images are reconstructed to extract the enhanced image. The flow diagram of the Laplacian Gaussian pyramid transform for the decomposition and reconstruction processes of image is shown as Figure 2. The original image is filtered by Gaussian low-pass filter and subsampled to produce *g*_1_. The image *g*_1_is then interpolated or made the convolution operations with kernel width 5 to reproduce the original array size and subtracted pixelwise from the original image to produce *b*_0_. This subband image *b*_0_, which is produced by an equivalent process of high-pass filter, is the finest level of the Laplacian Gaussian pyramid. The decimated low-pass filtering image *g*_1_ is further filtered by the Gaussian low-pass filter and subsampled producing *g*_2_. The *g*_2_ is interpolated or made the convolution operations with kernel width 5 and subtracted from the *g*_1_, which results in the second pyramid layer *b*_1_. All subsequent layers of the Laplacian Gaussian pyramid are computed by repeating these operations to the subsampled Gaussian low-pass filtering images from the previous iteration, until the setting pyramid level image *b*_*L*−1_ and the last level pyramid image *g*_*L*_ are obtained. The flowchart of the reconstruction process is drawn on the right hand of the Figure 2. The subimage *g*_*L*_ is interpolated or made the convolution operations with kernel width 5 to the array size of the next finer pyramid level *b*_*L*−1_, and the enhanced *b*_*L*−1_′ is added in this pixelwise to produce *g*_*L*−1_′. Interpolation, contrast enhancement, convolution, and addition operations are repeated until the reconstructed image at the original resolution level is obtained. The reconstruction is completely reversible if the interpolation filters used in decomposition and reconstruction are identical [10, 16–19].

It is obvious that features and contrast enhancement processes are implemented between the *b*_*L*−1_ and *b*_*L*−1_′. The image *g*_*L*_ is adjusted according to mathematical morphological operations to obtain contrast enhancement *g*_*L*_′. Repeating the reconstruction process can extract the enhanced image.

### 2.3. Contrast Limited Adaptive Histogram Equalization

Histogram equalization is a specific case of the more general class of histogram remapping methods. Histogram equalization, because of its advantages of high speed and better effect, is widely applied to enhance the contrast of mammographic images. Histogram is the function of gray level, which denotes the gray level of every pixel. Therefore, the contrast ratio will be improved by a gray nonlinear transform to adjust the accumulation function, and the gray in small range will be transformed in the whole field.

The histogram is a discrete function and defined as follows:

(1) $$\begin{array}{c} p_{r}\left(r_{k}\right)=\frac{n_{k}}{n}, \end{array}$$

where *n* is the total pixels of a mammogram and *n*_*k*_ is pixel number of corresponding the *r*_*k*_ gray level.

Hypothesis that the gray transfer function is *s* = *T*(*r*), whose slope is limited to non-minus continuum monotone increasing function, and it can transform input image *I*(*x*, *y*) to output image *I*′(*x*, *y*). Let *p*_*r*_(*r*) and *p*_*s*_(*s*) represent the probability density function of the random variable *r* and *s*, respectively. *r* is the gray level of the input image and *s* is the gray level of the output image. According to the definition of the histogram and the cumulate density function, the original image histogram and processed histogram areas are equal to

(2) $$\begin{array}{c} p_{s}\left(s\right)=p_{r}\left(r\right)\frac{dr}{ds}. \end{array}$$

Let *s* belong to [0, *L* − 1], the gray transfer function is expressed as follows:

(3) $$\begin{array}{c} s=T\left(r\right)=\left(L-1\right)\overset{r}{\underset{0}{\int }}p_{r}\left(w\right)dw, \end{array}$$

where *w* is an integral dummy variable.

According to the properties of integral, we can make the derivation of formula (3) to get a new equation as follows.

(4) $$\begin{array}{c} \frac{ds}{dr}=\frac{dT\left(r\right)}{dr}\left(L-1\right)\frac{d}{dr}\left[\overset{r}{\underset{0}{\int }}p_{r}\left(w\right)dw\right]=\left(L-1\right)p_{r}\left(r\right). \end{array}$$

Further, we can put the formula (4) into (2) and obtain a new formula as follows:

(5) ps(s)=pr(r)|drds|=pr(r)|1(L−1)pr(r)|=1L−1, 0≤s≤L−1.

According to (1) and the gray transfer function (3) transform, we can induce a discrete form given as follows:

(6) sk=T(rk)=(L−1)∑j=0kpr(rj)=L−1n∑j=0knj, k=0,1,2,…,L−1.

In generally, (6) is the gray level remapping function.

Comparing with the whole histogram equalization, the adaptive histogram equalization (AHE) has the advantage of good local contrast enhancement. But the AHE needs to compute the local histogram and accumulate distribution function of every pixel; it is extremely computational intensive. Besides, the AHE is sensitive to noise.

**Figure 3:**
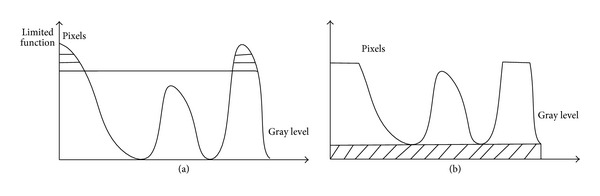
Limited function chart. (a) Original gray chart and (b) limited contrast gray chart.

The AHE enhances the image contrast and enlarges the noise. In some cases, enhancement process results in image distortion in some detail area, which affects the visual diagnosis of clinicians for the enhanced image [20, 21]. We need to not only enhance the features and image contrast, but also restrict the magnified noise. Thus limiting contrast function to AHE in every block is needed to generate transform function, respectively. How much percent of contrast will be restricted and enhanced? It is need to preliminarily adjust the contrast of each block at each pyramid level image. Thus, we define a limited function to limit the gray level probability density and control the exceed histogram. The adjustable processing is shown as Figure 3.

### 2.4. Mathematical Morphology

Mathematical morphology originated in set theory, geometry, and topology establishes the relationship between the geometry of physical system and some of its property. Most essential image processing algorithms can be represented in the form of morphological operations. For example, morphology offers a unified and powerful approach to different image processing problems [9, 11, 22]. We apply the morphological opening and closing operations to process the different multiscale subband images. The opening and closing operations are produced by combining dilation and erosion operator, respectively. Furthermore, a structuring element SE of rectangle shape is used in dilation and erosion operator, respectively.

The erosion of a gray-scale digital image *I*(*x*, *y*) by a structural element SE(*i*, *j*) is defined as follows [7, 9, 11].

(7) $$\begin{array}{c} \left(I⊗\text{SE}\right)\left(m,n\right)=\min\left\{I\left(m-i,n+j\right)-\text{SE}\left(i,j\right)\right\}. \end{array}$$

The gray-scale dilation can be described as

(8) $$\begin{array}{c} \left(I⊕\text{SE}\right)\left(m,n\right)=\max\left\{I\left(m-i,n-j\right)+\text{SE}\left(i,j\right)\right\}. \end{array}$$

The morphological opening and closing operations have the same form as the binary counterparts. The opening operation of image *I*(*x*, *y*), using the structure elements SE, is defined as erosion followed by dilation and expressed as (9). Utilizing the structure element SE, the closing operation of image *I*(*x*, *y*) is defined as dilation followed by erosion and given by (10) as follows:

(9) $$\begin{array}{c} I\circ \text{SE}=\left(I⊗\text{SE}\right)⊕\text{SE}, \end{array}$$

(10) $$\begin{array}{c} I•\text{SE}=\left(I⊕\text{SE}\right)⊗\text{SE}. \end{array}$$

The opening and closing operations can be interpreted as follows: the opening can remove all of the pixels (light details) in a region that are smaller than the structure element. For the corresponding opposite sequence, the closing can fill in holes and concavities smaller than the structure element. The two operations can remove the noise in the image and cannot make the image distortion.

In practical application morphological opening and closing pairs are combined in sequence for various image processing operations. There are two morphological operations known as top-hat (TH) and bottom-hat (BH) transformations. The top-hat transformation by opening is defined as the difference between the original image and its gray scale opening using structuring element SE and it is defined as

(11) $$\begin{array}{c} \text{TH}=I-\left(I\circ \text{SE}\right). \end{array}$$

Similarly dual bottom-hat transformation by closing is the difference between the gray-scale closing image and original image as described by

(12) $$\begin{array}{c} \text{BH}=\left(I•\text{SE}\right)-I. \end{array}$$

Based on the previous theoretical analysis, the TH transformation is an effective technique for enhancing small bright details from the background. Conversely, the dark features can be extracted from a brighter background by the BH transformation. In order to enhance the local contrast of the mammograms, the processing procedure is adding original image to the top-hat transformed image, and subtracting the bottom-hat image. Furthermore, its efficiency in image contrast enhancement has been proved by [9]. The calculated formula is given as follows:

(13) $$\begin{array}{c} C=I+\text{TH}-\text{BH}. \end{array}$$

Equation (13) has been used to process the low-pass frequency coefficients in the proposed algorithm, which enhances the features and contrast of mammographic image.

### 2.5. Global Gain Adjustment

We adopt a global gain adjustment technique in our investigation for histogram equalization enhancing part pixels uniformly at each pyramid level, which makes the visualization of the enhanced image to become more natural. We employ the mean introduced in [3, 22] to accomplish this nonlinear operation. The global gain adjustment function can be expressed as

(14) $$\begin{array}{c} f\left(z\right)=a\left[\text{sigm}\left(c\left(z-b\right)\right)-\text{sigm}\left(-c\left(z+b\right)\right)\right], \end{array}$$

where *a* = (1/(sigm(*c*(1 − *b*)) − sigm(−*c*(1 + *b*)))) subject to 0 < *b* < 1, *b* and *c* are ratio coefficients of enhancement and can be set different values according to the practical testing experiment to adaptively adjust.

The sigm(*z*) is described as follows:

(15) $$\begin{array}{c} \text{sigm}\left(z\right)=\frac{1}{1+e^{-z}}. \end{array}$$

## 3. Results and Discussion

The proposed algorithm has been applied to more than 10 mammographic images being 1944∗3072 in sizes from the Angell Medical ADM-600 MG. We had readjusted the size of each tested original image to be 1280∗3072 in order to decrease the cost times. The testing procedure has been implemented in MATLAB2012a. To demonstrate the effectiveness of our method, we compared its results with the existing popular methods of histogram equalization (HE), AHE, the algorithm of nonlinear multiscale processing based on Laplace pyramid proposed in [10], and the method based on morphology and wavelet transform. Furthermore, we take the advantage of the metrics of contrast evaluation criterion, SNR, and contrast improvement index (CII) to measure the quantitative performance analysis of the proposed method.

### 3.1. Contrast Evaluation Criterion for Image

The contrast of enhanced image is evaluated by employing the metric function, which was proposed in [4], and given as follows:

(16) $$\begin{array}{c} C_{c}=\frac{1}{MN}\overset{M}{\underset{i=1}{\sum }}\overset{N}{\underset{j=1}{\sum }}f^{\prime 2}\left(i,j\right)-{\left|\frac{1}{MN}\overset{M}{\underset{i=1}{\sum }}\overset{N}{\underset{j=1}{\sum }}f^{\prime }\left(i,j\right)\right|}^{2}, \end{array}$$

where *M* and *N* are height and width of the image, respectively, and *f*′(*i*, *j*) is the enhanced image. The larger the value of (16) is, the better the contrast of the image is.

### 3.2. Contrast Improvement Index

A quantization measure of contrast enhancement can be defined by a contrast improvement index, and its formula can be expressed by the following [8, 14, 15, 23]

(17) $$\begin{array}{c} \text{CII}=\frac{C_{\text{processed}}}{C_{\text{original}}}, \end{array}$$

where *C*_processed_ and *C*_original_ are the contrasts of the processed and original images, respectively. *C*is the average value of the local region contrast in the processed or original image. Thus, the CII value of original image is equal to one. The local contrast at each pixel is measured as (*X*_ max_ − *X*_ min _)/(*X*_ max_ + *X*_ min _) in its local window size. We applied a version of the optimized definition of contrast demonstrated in [8]. The contrast *C* of an image is described as follows:

(18) $$\begin{array}{c} C=\frac{m_{f}-m_{b}}{m_{f}+m_{b}}, \end{array}$$

where *m*_*f*_ is the mean luminance value of the foreground and the corresponding *m*_*b*_ is equal to the mean luminance value of background. In our experiment, we use the 5∗5 local windows. The greater value of CII indicates that the given image quality is better.

**Figure 4:**
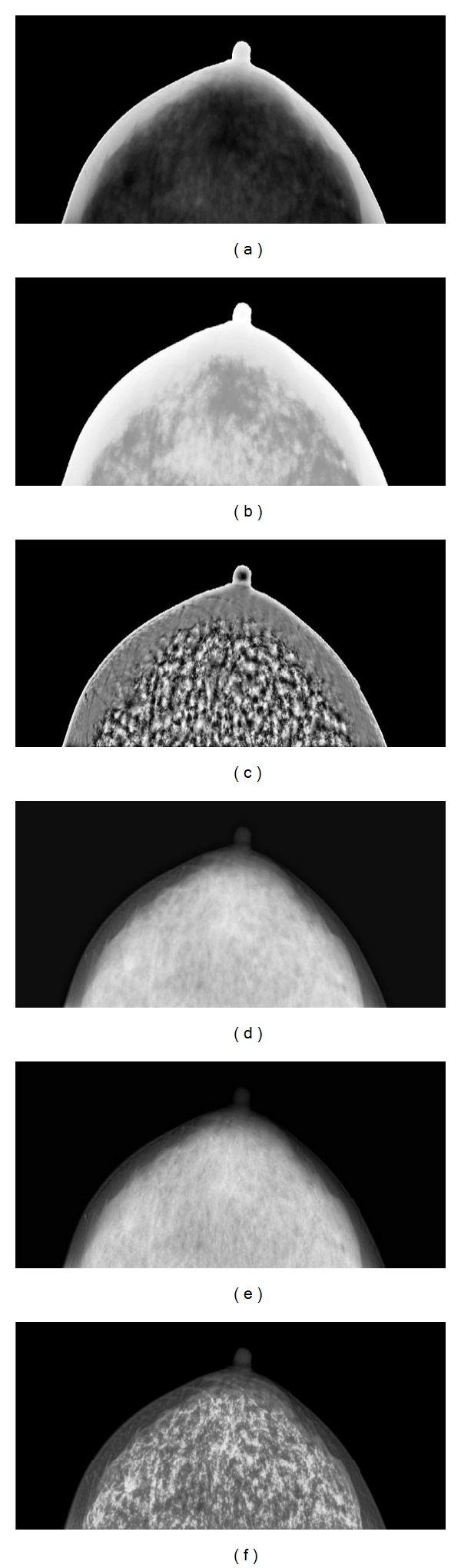
Comparison of contrast enhancement of mammograms. (a) Original mammogram. (b) Enhanced through histogram equalization. (c) Enhanced through adaptive histogram equalization. (d) Enhanced through nonlinear multiscale processing based on Laplace pyramid. (e) Enhanced through the method based on wavelet transform and morphology. (f) Enhanced through the proposed method.

**Figure 5:**
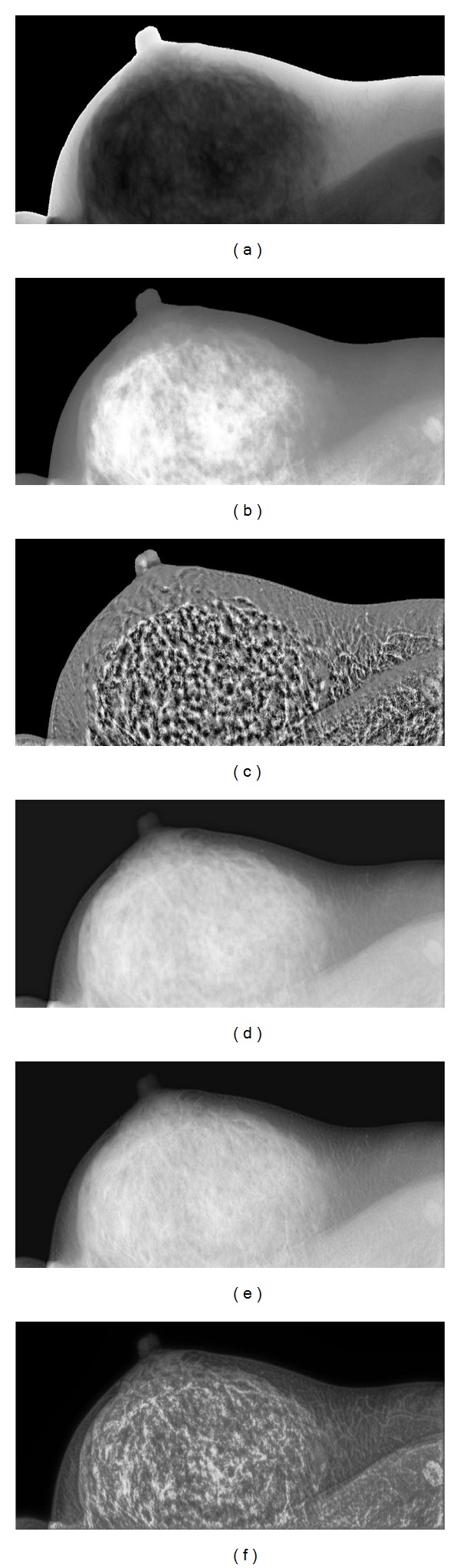
Comparison of contrast enhancement of mammograms. (a) Original mammogram. (b) Enhanced through histogram equalization. (c) Enhanced through adaptive histogram equalization. (d) Enhanced through nonlinear multiscale processing based on Laplace pyramid. (e) Enhanced through the method based on wavelet transform and morphology. (f) Enhanced through the proposed method.

As shown in Figures 4 and 5, (a) represents one of two different women's mammographic original images, respectively, and ((b)–(f)) are produced by HE, AHE, nonlinear multiscale processing based on Laplace pyramid, the method based on morphology and wavelet transform, and the proposed method, respectively. In the histogram equalization, the pixels are spreading uniformly. The traditional HE method is adopted to enhance the original image. The produced results through HE improve the contrast a little, but we find it difficult to identify tissue nodes and features, and the HE over amplifies the noise which results in the fibroglandular albefaction and the enhanced results cannot be applied to clinic. It seems that the enhanced contrast images through AHE method are degraded in visual quality. In addition, the AHE method over enhances the original images. The contrast and features of the enhanced images are improved by using Stahl's algorithm. However, it can be seen that the noise are enlarged. The low frequency components are ignored. It is difficult to identify the fibroglandular and tissue nodes, and the results cannot be applied in clinical application. Although the algorithm based on wavelet transform and morphology has made the original image become smoother and made the noise of image decrease and enhanced the mammograms contrast, but we can almost not distinguish the details and tissue nodes. Hence, the processed images do not play a significant role in practical clinic. The results enhanced by the proposed algorithm are not only saving good details but also the edges are preserved and enhanced. Features and fibroglandular are enhanced, the tissue nodes can be identified clearly in the apparent visual quality, and the contrast of mammogram has been improved a lot.

In Tables 1 and 2, comparison of values of contrast and contrast improvement index shows that the proposed method outperforms the HE, AHE, the approach of nonlinear multiscale processing based on Laplace pyramid, and the method based on wavelet transform and morphology in all the experimental images, which indicates better contrast enhancement of images. Corresponding to SNR values, the enhanced results of the wavelet method are the largest in our experiments. SNR is closely related to the luminance of image. The enhanced mammographic images of the wavelet method are the brightest in visualization, but the results do not play a significant role in the clinical application. We can clearly identify that the proposed method can better enhance image contrast and preserve the detailed information of the image. Both visual quality and quantitative measurements have shown that the proposed method is more suitable for features and contrast enhancement of mammographic images.

## 4. Conclusion

A method based on the Laplacian Gaussian pyramid transform, mathematical morphology, and contrast limited adaptive histogram equalization is proposed for features and contrast enhancement of mammographic images, which employs the penalty terms to adjust the various aspects of contrast enhancement. Thus, the proposed method enhances the image contrast at the same time as it effectively restricts the enlarged noise. The proposed method has been tested on mammograms and compared with the existing popular approaches of histogram equalization and adaptive histogram equalization, nonlinear multiscale processing based on Laplace pyramid, and the method based on wavelet transform and morphology. Furthermore, the experimental results show that the proposed method seems more suitable for mammographic images enhancement in both visual quality and qualitative measurements. Besides, the experimental results yielded by the proposed method still have a little unnatural in visualization. Therefore, the next step we will improve the proposed method and test it using mammographic images from the standard Database Mammographic Image Analysis Society (MIAS). For performance evaluation of the proposed algorithm, SNR, contrast evaluation criterion and contrast improvement index are adopted. The experimental results and metric data tables show that the proposed method seems to yield significantly the enhanced images. Comparing with other tested methods, the results of the proposed method are more suitable for clinic application.

## Figures and Tables

**Table 1:** Measurement evaluation of contrast, CII, and SNR for Figure 4.

Method	Contrast	CII	SNR
Original image	0.0503	1.0000	
HE	0.1607	2.5140	14.1344
AHE	0.0756	3.5981	4.0076
Nonlinear method based on Laplace pyramid	0.1230	4.0467	6.9851
Wavelet method	0.1137	5.4673	**18.1565**
The proposed method	**0.2677**	**14.5514**	12.9723

**Table 2:** Measurement evaluation of contrast, CII, and SNR for Figure 5.

Method	Contrast	CII	SNR
Original image	0.1140	1.0000	
HE	0.1232	1.5781	17.5618
AHE	0.0785	1.8958	4.5959
Nonlinear method based on Laplace pyramid	0.1254	1.8594	7.9491
Wavelet method	0.1205	2.6146	**20.6979**
The proposed method	**0.2141**	**7.0373**	14.6316
